# Expression of Programmed Cell Death-Ligands in Hepatocellular Carcinoma: Correlation With Immune Microenvironment and Survival Outcomes

**DOI:** 10.3389/fonc.2019.00883

**Published:** 2019-09-11

**Authors:** Haotian Liao, Wen Chen, Yunlu Dai, Joseph J. Richardson, Junling Guo, Kefei Yuan, Yong Zeng, Kunlin Xie

**Affiliations:** ^1^Liver Transplantation Division, Department of Liver Surgery, West China Hospital, Sichuan University, Chengdu, China; ^2^Department of Biological Chemistry and Molecular Pharmacology, Harvard Medical School, Boston, MA, United States; ^3^Faculty of Health Sciences, University of Macau, Macau, China; ^4^Department of Chemical and Biomolecular Engineering, ARC Centre of Excellence in Convergent Bio-Nano Science and Technology, University of Melbourne, Parkville, VIC, Australia; ^5^John A. Paulson School of Engineering and Applied Sciences, Wyss Institute for Biologically Inspired Engineering, Harvard University, Boston, MA, United States; ^6^Laboratory of Liver Surgery, West China Hospital, Sichuan University, Chengdu, China

**Keywords:** programmed cell death-ligands, macrophage, tumor immune stroma, tumor microenvironment, anti-PD-1 axis therapy, hepatocellular carcinoma

## Abstract

The quantity of programmed cell death-ligand 1 (PD-L1) is regarded as a predicting factor of clinical response to anti-PD-1 axis immunotherapy. However, the expression of PD-L1 and its prognostic value in hepatocellular carcinoma (HCC) patients remain debated. Meanwhile, the molecular features of PD-1's other ligand, namely PD-L2, as well as its correlation with clinicopathological parameters and HCC tumor microenvironment (TME), are still poorly understood. In this study, immunohistochemistry (IHC) data from 304 HCC patients were used to determine the clinicopathological features of PD-L1 and PD-L2 and their correlation with CD8^+^ T cells in HCC. Moreover, fresh clinical HCC samples were used to identify the immune cell subtypes expressing PD-L1 and PD-L2. By using The Cancer Genome Atlas (TCGA) dataset, we further assessed the correlation between mutation signature, copy number variation (CNV), number of neoepitopes, immune gene expression, immune/stromal cell infiltration to the expression of PD-L1 and PD-L2. While membrane expression of PD-L2 was observed in 19.1% of tumor samples, no obvious expression of PD-L1 was detected on tumor cell membranes. High expression of PD-L2 on tumor membranes and PD-L1 in immune stroma were both significantly associated with poorer overall survival (OS) and disease-free survival (DFS) outcomes. Flow cytometry analysis and immunofluorescence showed that macrophages were the main immune cell subtype expressing both PD-L1 and PD-L2. Moreover, positive expression of PD-Ls was correlated with higher CD8^+^ T cells infiltration in immune stroma. CNV analysis showed a similarity between PD-L1 and PD-L2 in affecting gene expression. In addition, higher levels of PD-Ls correlated with higher expression of immune related genes, enhanced cytolytic activity, and larger proportions of immune/stromal cell infiltration. Collectively, our study reveals the impact of both PD-L1 and PD-L2 on the HCC tumor microenvironment for the first time, providing insight for new therapeutic options.

## Introduction

Hepatocellular carcinoma (HCC) has recently become the second leading cause of cancer death around the world (1). Once diagnosed with HCC, only a small fraction of patients at early stages can benefit from curative treatments, including resection, local ablation or liver transplantation. While it has been reported that sorafenib can modestly extend the median survival of HCC patients at advanced stages (2), poor tolerance, and limited benefits due to relatively high drug resistance make the use of sorafenib a palliative care. Therefore, it's highly desirable to expand the therapeutic arsenal for HCC treatment.

Recently, the development of immune checkpoint inhibitors, which are capable of promoting the immune system to attack cancer cells, has experienced remarkable success (3). The interactions between the programmed death receptor 1 (PD-1) and programmed cell death-ligand 1 and 2 (PD-L1 and PD-L2) can induce T-cell exhaustion, which allows tumor cells to evade the immune system, leading to the failure of autoimmunity (4, 5). Thus, blocking PD-1 and PD-L1 association has become a key target for cancer immunotherapy. Two PD-1 inhibitors (Pembrolizumab and Nivolumab) and three PD-L1 (Atezolizumab, Avelumab, and Durvalumab) inhibitors have been approved, with others being tested in clinical trials at this moment.

Although the antitumor activity of PD-1 and PD-L1 inhibitors has been proved in various cancers (6–9), the clinical response to these therapies varies in different tumor types due to different PD-1 and PD-L1 behavior in individual patients. Therefore, significant efforts have been targeted toward finding predictive biomarkers to improve the identification of patients with potentially high therapeutic efficiency. The expression of PD-L1 in tumors, a main factor to determine the eligibility of patients for PD-1 axis targeted treatments, is conventionally detected by immunohistochemistry (IHC). However, a large proportion of PD-L1-positive patients have responded poorly to anti-PD-1 axis therapies, while some PD-L1-negative patients have unexpectedly shown favorable therapeutic responses (10–14), suggesting that molecular interactions involving PD-1 independent from PD-L1 could also serve as predicting factors in the clinical responsiveness to these therapies. As another PD-1 ligand, PD-L2 is also commonly detected in human tumors, especially with its expression observed in the absence of PD-L1. Therefore, PD-L2 holds great value for predicting clinical response to anti-PD-1 axis therapies (11, 15–17). The expression of PD-L1 in HCC patients and its correlation with clinicopathological parameters have been fully illustrated. However, investigation of PD-1/PD-L2 interactions in HCC is still lacking (18, 19). The molecular features of both ligands, and their correlation with clinicopathological parameters and the tumor microenvironment (TME), are still poorly understood.

In this study, we aimed to define the biological and clinical relevance of the expressions of PD-L1 and PD-L2, as well as their correlation with each other in HCC. We used tissue microarrays (TMA) to examine 304 HCC tumor samples and analyzed the genomics data of HCC patients from The Cancer Genome Atlas (TCGA). The clinical and histopathological features of PD-L1 and PD-L2 in HCC tumor samples were assessed by IHC. Their precise locations in immune cell subtypes were detected by flow cytometry and immunofluorescence. Moreover, we evaluated how PD-L1 and PD-L2 expression are associated with transcriptomic profiles, copy number variation (CNV), and somatic mutations. These findings warrant further study focusing on both PD-L1 and PD-L2 as biomarkers of clinical outcomes to PD-1 blockade in HCC patients. Meanwhile, our work also provided a rationale for developing more effective anti-PD-1 axis immunotherapies for HCC.

## Materials and Methods

### Patients and Samples

A total of 304 HCC patients undergoing hepatectomy between 2007 and 2012 in West China Hospital were included in this study. Tissue cores punched from representative tissue areas of formalin-fixed, paraffin-embedded (FFPE) HCC samples were selected to the construct tissue microarray (TMA). Tumor staging classification was carried out according to the 7th AJCC TNM Staging for Liver and Intrahepatic Bile Duct Malignancies. The characteristics of tumor samples including differentiation, size, number of nodules, vascular invasion, and Ishak fibrosis score of the adjacent liver tissue were evaluated by two pathologists specializing in hepatic diseases. The primary end point of this study was overall survival (OS), which was defined as the time from the date of surgery to the date of death without regard to the cause of death. The secondary end point was disease-free survival (DFS) defined as the time from the date of surgery to the time of the first event (recurrence, progression, death). Patients with no events were censored at the date of their last follow-up. Informed consent for the use of tissue for the purpose of biomedical research was provided by every individual patient, with the approval from the ethics committee of West China Hospital.

### Immunohistochemistry

TMA tissue sections were deparaffinized in xylene and rehydrated by sequential incubation in EtOH/water solutions. Antigen retrieval was conducted by heating the sections under high pressure in EDTA antigen retrieval solution against antibodies PD-L1 (1/400, Cell Signaling Technology), PD-L2 (1/400, R&D Systems), and CD8 (1/100, Abcam). The sections were incubated overnight at 4°C for primary antibodies. Then peroxidase reagents were used to limit steric interference and provide enhanced accessibility of secondary antibodies to the target. Envision system (enzyme-conjugated polymer backbone coupled to secondary antibodies) and 3, 3′-diaminobenzidine (DAB) chromogen were applied to perform the staining. Placenta tissues were used as a positive control for both PD-L1 and PD-L2 expressions. Negative controls included the use of PBS instead of primary antibodies, with all other conditions preserved. TMA images were viewed and captured using the NDP.view.2 software program. Slides were reviewed by two experienced pathologists who were blind to the clinical parameters. The expressions of PD-L1, PD-L2 were evaluated in tumor cells and tumor-infiltrating immune cells in HCC stroma. Expression of PD-L1 in immune stroma were recorded as the total number of cells expressing the protein in five respective areas at 40× magnification from each immunostained HCC section. Positive staining of PD-L1 in stroma was defined as the number of positive cells being higher than the median of the full series. The same strategy was used to record the expression of PD-L2 and CD8 in stroma. Positive staining of PD-L1 and PD-L2 in tumor cells was verified if staining of more than the membrane/surface was observed on over 1% of the tumor cells (20, 21).

### Immunofluorescence

Immunofluorescence (IF) staining was performed on FFPE HCC tissue samples as previously described (22). The used antibodies are listed as follows, anti-PD-L1 (1,100, Cell Signaling Technology), anti-PD-L2 (1,100, R&D system), anti-CD8 (1,100, Abcam), and anti-CD68 (1,50, Santa Cruz). The corresponding secondary antibodies (Molecular Probes) were applied sequentially, and DAPI (Sigma) was used for nuclear staining. All images were acquired via Zeiss Axio Imager.

### Flow Cytometry

Peripheral blood samples from untreated patients with pathologically confirmed HCC were obtained before curative resection. After surgical resection of the HCC, fresh tumor samples and adjacent normal tissues were taken and cut into small pieces, then enzymatically digested with 0.05% collagenase IV (Sigma-Aldrich), 0.002% DNase I (Roche), and 20% FBS (HyClone Laboratories) in RPMI1640 medium (Gibco) at 37°C for 20 min. The resulting suspension was passed through 70 μm cell strainers (BD bioscience). The red blood cells in peripheral blood, HCC, and adjacent normal tissues were lysed using 1× Lysing Buffer (BD bioscience). The following fluorophore conjugated antibodies were used for flow cytometry, anti-CD3 (APC, BD bioscience), anti-CD4 (Alexa Fluor 700, BD bioscience), anti-CD8 (APC cy7, BD bioscience), anti-CD45RO (BV510, BD bioscience), anti-HLA-DR (PE-cy5, BD bioscience), anti-CD14 (FITC, BD Biosciences), anti-lin1 (FITC, Biolegend), anti-CD11c (APC, BD Biosciences), anti-CD123 (PE-cy7, BD Biosciences), anti-PD-L1 (BV421, BD Biosciences), and anti-PD-L2 (PE, BD Biosciences). Cells were incubated with antibodies in the dark for 30 min at 4°C in PBS + 1% BSA + 10% FBS. An aqua live/dead viability stain (Invitrogen) was added before flow cytometry analysis using the BD FACSDiva software. In order to relieve the effect of non-specific background signal when distinguishing PD-L1^+^ and PD-L2^+^ macrophages, various controls, including negative controls, isotype controls, and single staining controls (only one type of PD-Ls antibody was added, either PD-L1 or PD-L2), were used in our study.

### Statistical Analysis

The significance of associations between PD-L1 and PD-L2 expression, as well as the associations between PD-Ls and CD8 expression, were assessed by the χ^2^ test. Survival rates of expression level (low vs. high) were estimated by the Kaplan-Meier method with Rothman CIs. Survival curves were compared with the log-rank test. The hazard ratio (HR) and 95% CI associated with the expressions of PD-L1, PD-L2, and CD8 were estimated through a multivariable Cox regression model adjusted for TNM stage (I vs. II-III), HBV infection (no vs. yes), tumor size ( ≤ 5 vs. >5 cm) and microvascular invasion (no vs. yes). Statistical analyses and graphics were conducted using R version 3.3.2.

## Results

### PD-Ls Expression and Correlation With Clinicopathological Features of HCC Patients

**Figure 1:**
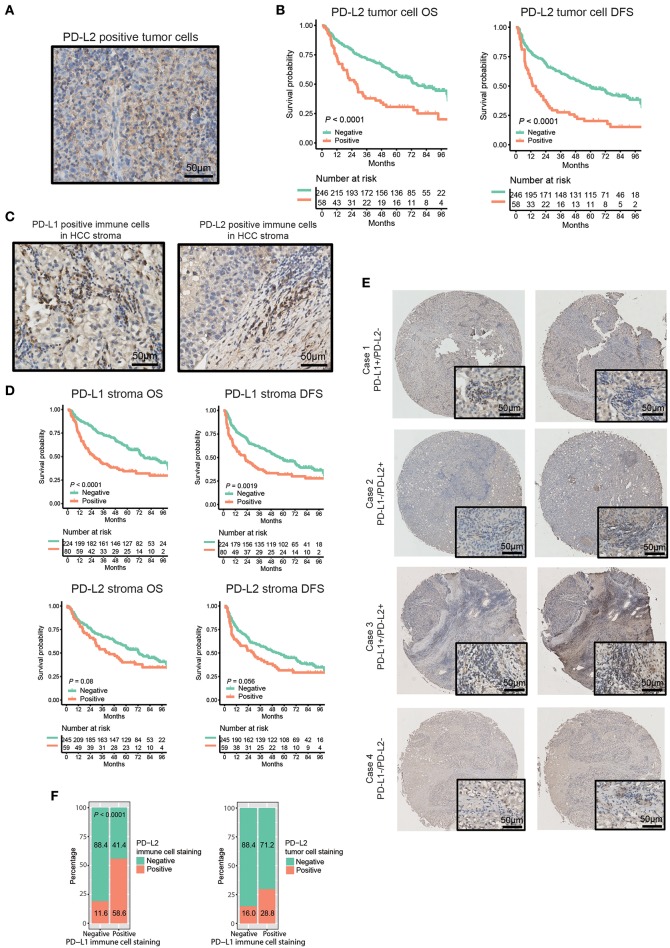
Distribution and expression of PD-Ls and their correlation with prognosis in HCC patients. **(A)** IHC staining of PD-L2 on tumor cell membrane. **(B)** Kaplan-Meier curve of OS and DFS stratified by PD-L2 expression on tumor cell membrane. **(C)** IHC staining of PD-L1 and PD-L2 in immune stromal cells. **(D)** Kaplan-Meier curve of OS and DFS stratified by PD-L1, PD-L2 expression in immune stroma. **(E)** Representative images showing the association between expression of PD-L1 and PD-L2 in immune stroma. **(F)** Proportions of HCC tumor samples expressing PD-L2 in immune stroma (left panel) and on tumor cell membranes (right panel) stratified by PD-L1 expression in immune stroma.

The characteristics of patients are listed in Table 1. The median age of the patients was 49 years (ranging from 18 to 79 years old). Patients were dominated by male (82.9%) and a large proportion of patients were HBV-positive (84.9%). The presence of PD-L1 and PD-L2 in either tumor cell membranes or immune stroma was observed in 80 (26.3%) and 101 (33.2%) of the tumor samples, respectively. To our surprise, while membrane expression of PD-L2 was observed in 58 (19.1%) of tumor samples (Figure 1A, Table 1), no obvious expression of PD-L1 was detected on tumor cell membranes. Moreover, positive status of PD-L2 on tumor cell membranes was significantly associated with poorer OS and DFS (Figure 1B, both *P* < 0.0001). Regarding the expression in stromal immune cells, both PD-L1 and PD-L2 were detected in 80 (26.3%) and 59 (19.4%) of the tumor samples, respectively (Figure 1C, Table 1). While the expressions of PD-L1 in immune stroma were associated with poorer OS/DFS outcomes (Figure 1D, upper panel, *P* < 0.0001 and *P* = 0.0019, respectively), no statistically significant correlation was found between PD-L2 expression in immune stroma and OS/DFS outcomes (Figure 1D, lower panel, *P* = 0.08 and *P* = 0.056, respectively). Moreover, there are HCC samples exhibiting discordance between PD-L1 and PD-L2 in immune stroma, in which some displayed PD-L1 expression in the absence of PD-L2, while the rest showed the opposite (Figure 1E, Table 1). Nevertheless, a positive correlation was observed between PD-L1 and PD-L2 expressed by stromal immune cells (Figure 1F, left panel, *P* < 0.0001).

**Table 1:** Baseline characteristics of HCC patients.

**Characteristic**	**Total *N* = 304**
**Male**	252
**Age, Median (range), y**	49 (18–79)
**HBV infection**	
No	46
Yes	258
**TNM stage**	
I	141
II–III	163
**Tumor differentiation**	
Well	172
Poor	132
**Tumor size, cm**	
≤ 5	160
>5	144
**Tumor multiplicity**	
Single	246
Multiple	58
**Microvascular invasion**	
No	229
Yes	75
**Portal vein thrombosis**	
No	260
Yes	44
**Liver cirrhosis**	
No	115
Yes	189
**α-Fetoprotein, ng/mL**	
≤ 20	113
>20	191
**PD-L1 in stroma**	
Negative	224
Positive	80
**PD-L2 in stroma**	
Negative	245
Positive	59
**PD-L2 in tumor cell**	
Negative	246
Positive	58
**CD8 TIL**^**+**^ **in stroma**	
Negative	176
Positive	128
**PD-L1**^**+**^**/PD-L2**^**−**^ **in stroma**	47
**PD-L1**^**−**^**/PD-L2**^**+**^ **in stroma**	26
**PD-L1**^**−**^**/PD-L2**^**−**^ **in stroma**	198
**PD-L1**^**+**^**/PD-L2**^**+**^ **in stroma**	33

The association of combined PD-Ls expression in immune stroma with survival outcomes was also investigated (Figure S1). Significant differences in OS and DFS were found between patients with neither PD-L1 nor PD-L2 expressions and the other two groups of patients (PD-L1^+^PD-L2^−^/PD-L1^−^PD-L2^+^ and PD-L1^+^PD-L2^+^, respectively). Further statistical analysis demonstrated that there was a significant difference in the prevalence of PD-L2 on tumor cell membranes between PD-L1-positive and -negative samples (Figure 1F, right panel, *P* = 0.013). In addition, a strong correlation was observed between the mRNA expression levels of PD-L1 and PD-L2 in TCGA and GEO datasets (Figure S2). These findings suggested the high frequency of the co-existence of PD-L1 and PD-L2 in HCC. On the other hand, multivariate analysis showed that both PD-L1 in immune stroma and PD-L2 on tumor cell membranes were independent risk factors for OS and DFS (Table 2).

**Table 2:** Cox proportional hazard model showing hazard ratios for survival outcomes conferred by variables.

**Variables**	**Overall survival**				**Disease-free survival**			
	**Univariate**		**Multivariate**		**Univariate**		**Multivariate**	
	**HR(95% CI)**	***P*-value**	**HR(95% CI)**	***P*-value**	**HR(95% CI)**	***P*-value**	**HR(95% CI)**	***P*-value**
**Sex**								
Male	1(Ref)	0.479			1(Ref)	0.505		
Female	0.862(0.571–1.301)				0.877(0.596–1.290)			
**Age**								
≤ 60	1(Ref)	0.336			1(Ref)	0.246		
>60	1.165(0.854–1.589)				1.189(0.887–1.594)			
**HBV infection**								
No	1(Ref)	0.311			1(Ref)	0.723		
Yes	0.8149(0.549–1.210)				0.932(0.633–1.373)			
**TNM stage**								
I	1(Ref)	<0.0001	1(Ref)	0.012	1(Ref)	0.0001	1(Ref)	0.051
II-III	2.073(1.508–2.848)		1.642(1.114–2.419)		1.773(1.320–2.380)		1.442(0.999–2.082)	
**Tumor differentiation**								
Well	1(Ref)	0.106			1(Ref)	0.184		
Poor	1.285(0.945–1.741)				1.215(0.911–1.619)			
**Tumor size, cm**								
≤ 5	1(Ref)	<0.0001	1(Ref)	0.020	1(Ref)	0.0016	1(Ref)	0.088
> 5	1.907(1.403–2.594)		1.509(1.067–2.134)		1.587(1.190–2.116)		1.299(0.961–1.757)	
**Tumor multiplicity**								
Single	1(Ref)	0.003	1(Ref)	0.388	1(Ref)	0.013	1(Ref)	0.588
Multiple	1.719(1.204–2.455)		1.188(0.804–1.755)		1.537 (1.093–2.161)		1.108(0.763–1.608)	
**Microvascular invasion**								
No	1(Ref)	0.001	1(Ref)	0.404	1(Ref)	0.0008	1(Ref)	0.163
Yes	1.767(1.260–2.478)		1.184(0.796–1.762)		1.721(1.250–2.370)		1.307(0.897–1.904)	
**Portal vein thrombosis**								
No	1(Ref)	0.009	1(Ref)	0.248	1(Ref)	0.078		
Yes	1.712(1.145–2.558)		1.301(0.833–2.031)		1.421(0.961–2.101)			
**Liver cirrhosis**								
No	1(Ref)	0.586			1(Ref)	0.419		
Yes	1.029(0.749–1.413)				1.132(0.838–1.529)			
**α-Fetoprotein, ng/mL**								
≤ 20	1(Ref)	0.119			1(Ref)	0.157		
>20	1.292(0.936–1.785)				1.243(0.919–1.682)			
**PD-L1 in stroma**								
Negative	1(Ref)	<0.0001	1(Ref)	<0.0001	1(Ref)	0.002	1(Ref)	
Positive	1.992(1.436–2.763)		2.326(1.644–3.289)		1.632(1.192–2.233)		1.785(1.284–2.480)	0.0005
**PD-L2 in stroma**								
Negative	1(Ref)	0.081			1(Ref)	0.057		
Positive	1.384 (0.960–1.996)				1.399(0.989–1.979)			
**PD-L2 in tumor cell**								
Negative	1(Ref)	<0.0001	1(Ref)	0.0001	1(Ref)	<0.0001	1(Ref)	<0.0001
Positive	2.077(1.465–2.944)		2.027(1.407–2.921)		2.234(1.605–3.110)		2.244(1.587–3.172)	
**CD8**^**+**^ **TIL in stroma**								
Negative	1(Ref)	0.012	1(Ref)	0.0002	1(Ref)	0.011	1(Ref)	0.0008
Positive	0.666(0.485–0.914)		0.532(0.380–0.743)		0.682(0.506–0.918)		0.5880.430–0.803)	

### PD-Ls Expression in Immune Cell Subsets of Human HCC

**Figure 2:**
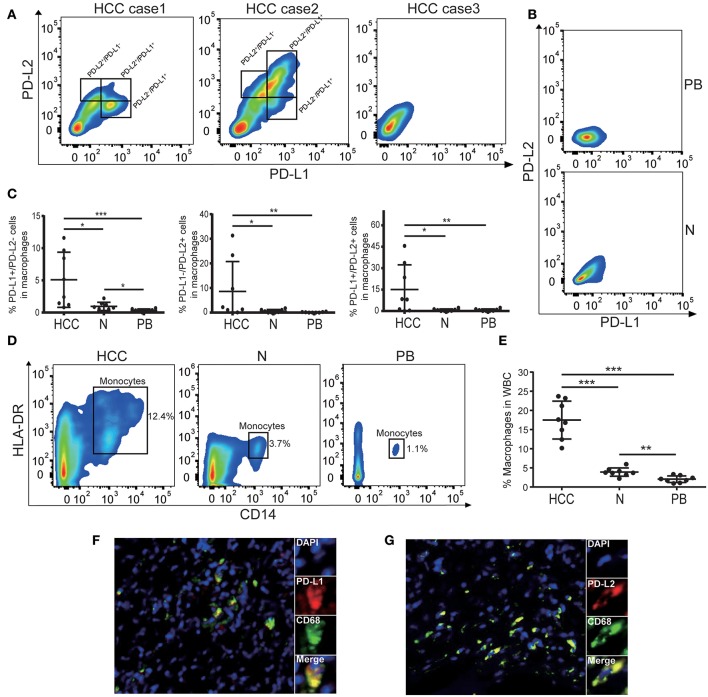
The expression of PD-Ls in macrophages from HCC tissues. **(A)** Representative flow cytometry analysis showing that both PD-L1 and PD-L2 can be expressed by HLA-DR^+^CD14^+^ macrophages. **(B)** Representative flow cytometry analysis demonstrating the absence of PD-Ls in HLA-DR^+^CD14^+^ macrophages in adjacent normal tissues and peripheral blood. PB, peripheral blood; N, adjacent normal tissues. **(C)** Aggregate data showing higher proportions of PD-L1^+^, PD-L2^+^, PD-L1^+^/PD-L2^+^ macrophages in HCC tissues compared with adjacent normal tissue and peripheral blood (*n* = 8). PB, peripheral blood; N, adjacent normal tissue; *P < 0.05; **P < 0.01; ***P < 0.001. **(D)** Flow cytometry plots demonstrating higher percentage of macrophages in HCC tissues compared with adjacent normal tissue and peripheral blood (*n* = 8). PB, peripheral blood; N, adjacent normal tissue. **(E)** Aggregate data showing a higher proportion of macrophages in HCC tissues compared with adjacent normal tissue and peripheral blood (*n* = 8). PB, peripheral blood; N, adjacent normal tissue; **P < 0.01; ***P < 0.001. **(F,G)** Immunofluorescence staining showing PD-L1^+^ **(F)** and PD-L2^+^ **(G)** in macrophages.

Previous study has shown that macrophages were the predominant PD-L1^+^ population of immune cells in the HCC microenvironment (23), however the subtype of immune cells expressing PD-L2 in HCC remains unknown. It has been reported that PD-L2 can be expressed by various immune cell subtypes in response to microenvironmental stimulation, including two major antigen-presenting cells (APC), dendritic cells (DCs), macrophages, and T cells (24–28). Therefore, we analyzed the prevalence of PD-L2 expression in these immune cell subtypes and its correlation with PD-L1. The results showed the enrichment of CD4^+^ and CD8^+^ T cells in HCC tissue (Figure S3A). While lin^−^HLA-DR^+^ CD4^+^ CD11c^+^ CD123^−^ myeloid dendritic cells (mDCs) could be detected, lin-HLA-DR^+^ CD4^+^ CD11c^−^ CD123^+^ plasmacytoid dendritic cells (pDCs) were absent in HCC tissue (Figure S3B). Regarding the immune cell subtypes expressing PD-Ls, no obvious expression of PD-L1 or PD-L2 was observed in CD4^+^ T cells, CD8^+^ T cells, or mDCs (Figures S3A,B). However, both PD-L1 and PD-L2 could be found in HLA-DR^+^CD14^+^ macrophages (Figure 2A). Together with the previous study (23), our findings indicated that macrophages were the predominant population expressing PD-Ls in HCC microenvironment. In some cases, Neither PD-L1 nor PD-L2 were presented in macrophages (Figure 2A). Moreover, the percentages of PD-L1^+^ PD-L2^−^, PD-L1^−^ PD-L2^+^, and PD-L1^+^ PD-L2^+^ macrophages were all higher in tumor tissues than adjacent normal tissue and peripheral blood (Figures 2A–C). Meanwhile, the results demonstrated that macrophages were more numerous in HCC tissues than in the corresponding peripheral blood and adjacent normal tissues, suggesting the aggregation of PD-Ls^+^ macrophages in tumor tissue (Figures 2D,E). Further validation by immunofluorescence also showed that both PD-L1 and PD-L2 could be expressed by macrophages in stroma (Figures 2F,G). These results indicated that there was an accumulation of tumoral macrophages expressing both PD-L1 and PD-L2, potentially contributing to carcinogenesis in the HCC microenvironment.

### CD8^+^ T Cell Infiltration in HCC and Its Association With the Expression of PD-Ls

**Figure 3:**
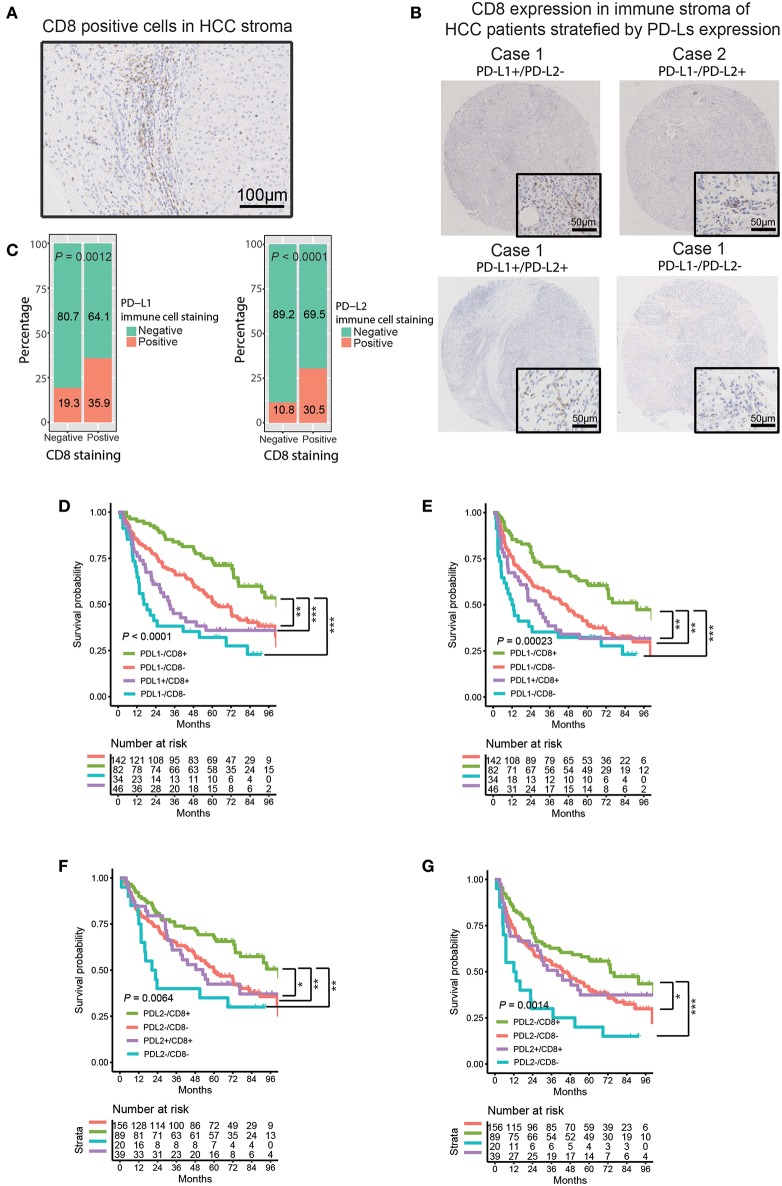
Correlation between PD-Ls and CD8^+^ T cell infiltration in HCC immune stroma. **(A)** Representative images showing CD8 expression in HCC immune stroma. **(B)** Representative CD8 expression in immune stroma of HCC patients stratified by PD-Ls expression. **(C)** Proportions of HCC tumor samples expressing CD8 stratified by PD-L1 and PD-L2 expressed by immune stroma. **(D–G)** Kaplan-Meier curves of OS **(D, E)** and DFS **(F, G)** stratified by PD-L1 (left panel) and PD-L2 (right panel) in combination with CD8 expression in immune stroma. *P < 0.05; **P < 0.01; ***P < 0.001.

Anti-PD-1 therapy accomplishes antitumor activity by blocking PD-1 in effector immune cells (such as CD8^+^ T cells) from interacting with their ligands, PD-L1/PD-L2 (29, 30). Most patients who benefited from anti-PD-1 therapy tended to have CD8^+^ T cell infiltration and higher PD-L1 expression in tumor tissue, and the expression of PD-L1 was significantly associated with CD8 density (31). Moreover, it has been reported that PD-L1 was predominantly expressed in the lymphoepithelioma-like subtype of HCC (LEL-HCC), which is characterized by sheets of neoplastic cells intermingled with a dense immune stroma made of cytotoxic T (CD8^+^) cells (20, 32, 33). These findings suggested a potential interaction between PD-Ls and CD8^+^ T cell infiltration in HCC immune stroma. Thus, we evaluated the association between CD8 and PD-Ls (both PD-L1 and PD-L2) in HCC immune stroma. Consistent with the previous studies (20, 32, 33), a similar pattern of CD8^+^ T cell infiltration in immune stroma was found in 128 (42.1%) tumor samples (Figure 3A, Table 1). Moreover, we found that patients with positive PD-L1 and/or PD-L2 expression tended to be of CD8 positive status, suggesting the co-existence of PD-L1^+^, PD-L2^+^ macrophages, and CD8^+^ T cells. Representative images from four cases were shown to illustrate the correlation between PD-L1, PD-L2 status, and CD8 expression in immune stroma (Figure 3B). Indeed, analysis on the protein expression in immune stroma (Figure 3C, PD-L1 vs. CD8, *P* = 0.0012; PD-L2 vs. CD8, *P* < 0.0001) and the mRNA level from TCGA and GEO datasets (Figure S4) showed that both PD-L1 and PD-L2 were positively associated with CD8 expression. Survival curve analysis showed that there was a positive correlation of CD8 expression with superior OS/DFS outcomes (Figure S5, Table 2). Moreover, the OS and DFS outcomes of PD-L1^−^/CD8^+^ patients were significantly better than other groups (Figures 3D,E), and PD-L1^+^/CD8^−^ status predicted poorer survival outcomes when compared with other groups (Figures 3D,E). These results were similar to what was found when stratifying patients into different subgroups based on PD-L2/CD8 expression, in which PD-L2^−^/CD8^+^ patients had a relatively better prognosis than other groups, while the prognosis of PD-L2^+^/CD8^−^ patients was poorer (Figures 3F,G).

### Mutational Event and CNV Analysis in Patients With High vs. Low Expression of PD-Ls

**Figure 4:**
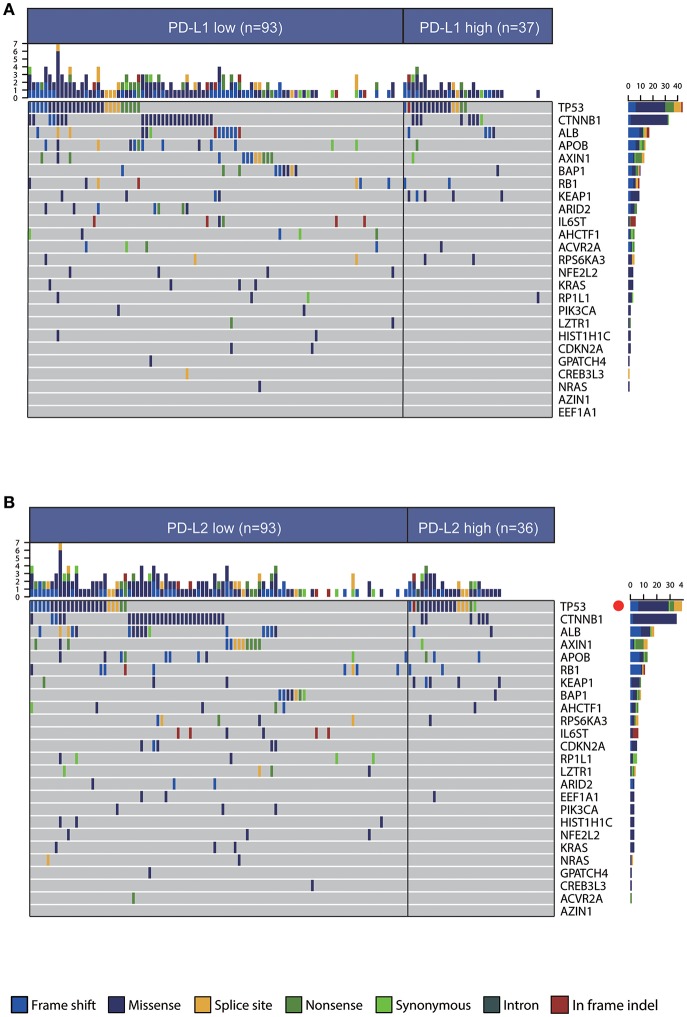
Association between PD-Ls and mutational signatures in HCC. **(A,B)** Significantly mutated genes in HCC subsets stratified by PD-L1 **(A)** and PD-L2 **(B)** expression. Red dot, *P* < 0.05.

An accurate landscape of the mutations in HCC driver genes has been provided by previous genetic profiling studies (34). Therefore, we sought to evaluate whether PD-Ls correlated with distinct mutational profiles characterized for HCC (Supplementary Materials). While no statistical difference could be identified in mutations between tumors with high and low PD-L1 expression, PD-L2-high tumors had a slightly higher mutation burden in TP53 (Figures 4A,B, χ^2^ test, *P* < 0.05). In the meantime, most mutations across the dataset were associated with G>A and C>T transitions, and higher frequencies of A>G and T>C transitions were observed in tumors low in PD-L1 (Figure S6A). However, the frequency of specific substitutions did not differ between PD-L2-high and -low tumors (Figure S6B).

**Figure 5:**
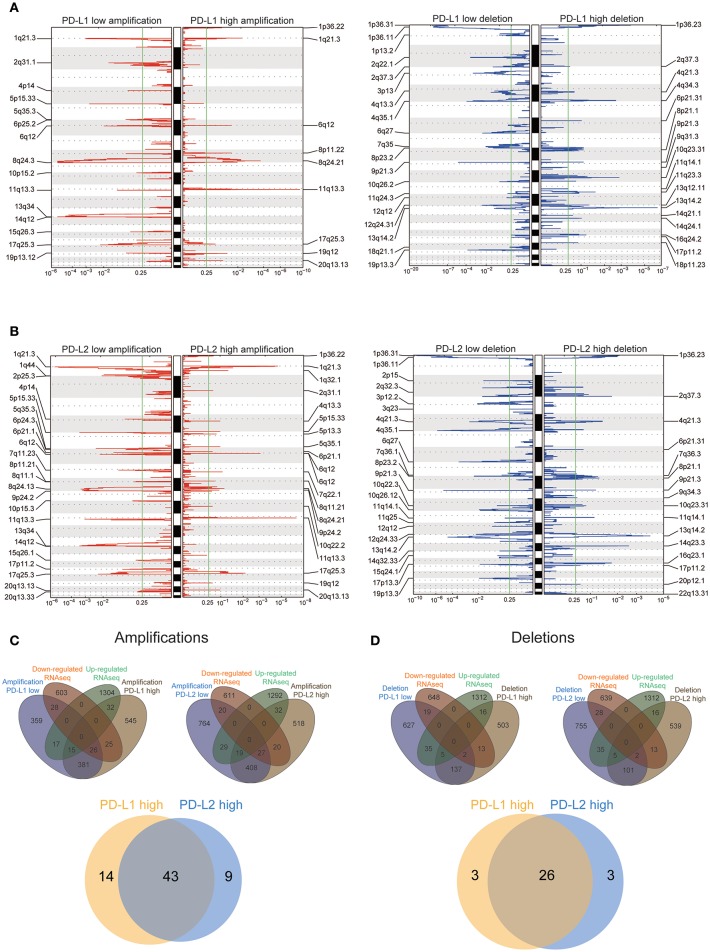
Association between PD-Ls and CNV in HCC. **(A,B)** GISTIC2.0 analysis identified recurrent somatic copy number alterations in different HCC subsets stratified by PD-L1 **(A)** and PD-L2 **(B)** expression. **(C,D)** Venn diagrams demonstrating the number of genes within genomic regions showing significant amplification or deletion, as well as the overlay with significant genes identified from RNAseq in different HCC subsets stratified by PD-L1 **(C)** and PD-L2 **(D)** expression. Each circle in the Venn diagram represents one set and the number in the overlaid area represents the common genes between the sets.

Previous studies have demonstrated increased genomic instability with extensive CNV in HCC patients (34–37), so we then assessed CNV between HCC subtypes with different expression of PD-Ls through GISTIC 2.0 analysis (Supplementary Materials). We discovered that a large number of cytobands were either significantly amplified or deleted regardless of the influence by PD-L1 or PD-L2 expression (Figures 5A,B). Both PD-L1-high and PD-L2-high subjects had 1024 genes within the chromosome regions with significant copy number amplification. After overlaying these genes with the differentially expressed genes identified by RNAseq, PD-L1-high and PD-L2-high subgroups had 57 and 52 genes within the amplification regions showing a concordant expression pattern in RNAseq, respectively (Figure 5C, upper panel). This implied that the different expression patterns of these genes were partially owing to copy number amplification. On the other hand, the numbers of genes within the deletion regions in PD-L1-high and PD-L2-high subgroups were both 676. Meanwhile, both PD-L1-high and PD-L2-high subgroups had 29 genes within the deletion regions showing a concordant expression pattern in RNAseq, suggesting that different expression patterns of these genes could partially due to the copy number deletion (Figure 5D, upper panel). In addition, for genes with different expression patterns owing to copy number amplification, there was an overlap between PD-L1-high group and PD-L2-high group (43 genes, Figure 5C, lower panel). Such behavior was similar to genes that attributed their different expression pattern to copy number deletion (26 genes, Figure 5D, lower panel). These findings suggested the potential similarity between the up-regulations of PD-L1 and PD-L2 in affecting CNV, which finally leads to the differential expression of these genes.

### Correlation Among Neoepitope Loads, Cytolytic Activity, Cytokine Gene Expressions, and the Expression of PD-Ls

**Figure 6:**
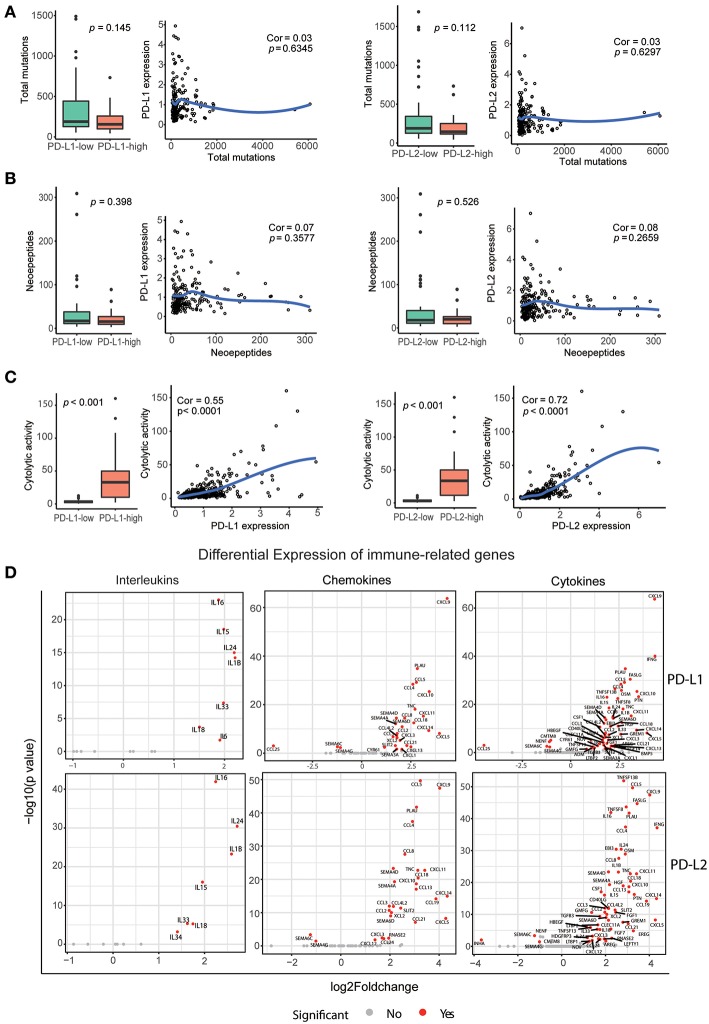
Correlation between PD-Ls and neoepitope loads, cytolytic activity, and immune gene expression. **(A)** Local regression curves (Spearman rank correlation) between expression of PD-Ls and total mutation count; boxplot distributions between HCC subsets stratified by the expression of PD-Ls (Mann-Whitney). **(B)** Local regression curves between the expression of PD-Ls and numbers of neoepitopes and boxplot distributions between HCC subsets stratified by the expression of PD-Ls. **(C)** Local regression curves between the expression of PD-Ls and cytolytic activity and boxplot distributions between HCC subsets stratified by PD-Ls expression. **(D)** Differentially expressed cytokines between PD-Ls-high and -low HCC samples.

Neoepitopes are peptides that arise from somatic mutations and are entirely absent from the normal human genome. They are reported to preferentially drive the T cell recognition of tumor cells (38). Therefore, we determined whether PD-Ls correlated with neoepitope loads in HCC (Supplementary Materials). This analysis revealed no correlation between total mutations per individual tumor and expression of PD-Ls (Figure 6A). Meanwhile, when viewed as a group, tumors with higher expression of PD-Ls exhibited no increase in the number of total mutations when compared to those with lower expression of PD-Ls (Figure 6A). In regard to neoepitope loads, no association was found between neoepitope loads and the expression of PD-Ls (Figure 6B). These findings suggested that the expression of PD-Ls is not driven by increased mutations or neoepitope loads.

Previous studies have provided evidence for immunoediting in tumors and have uncovered the mechanisms of tumor-intrinsic resistance to cytolytic activity by using large-scale genomic data sets of solid tissue tumor biopsies (36, 39). In this study, we also analyzed the potential of PD-Ls expression in affecting cytolytic activity. Cytolytic activity was defined as the geometric mean of GZMA and PRF1 (expressed in TPM, Supplementary Materials). We found that there is a strong correlation between cytolytic activity and the expression of PD-Ls (both PD-L1 and PD-L2) in HCC patients (Figure 6C), suggesting that immune response in cytolytic-high HCC samples can elicit tumor mechanisms of immune suppression regulated by PD-Ls.

Increasing evidence suggests that pro-inflammatory and anti-inflammatory cytokine imbalances are widely in existence in the tumor microenvironment. The presence of such imbalances promote the development and progression of HCC (40, 41). Moreover, it has been determined that engagement of PD-1 by PD-Ls dramatically affects T cell receptor (TCR)-mediated and cytokine production (24, 42). We hypothesized that expression of these cytokines, chemokines, and interleukins are dysregulated in HCC tumors with higher expression of PD-Ls (Supplementary Materials). Consistently, we found that the expression of various pro- and anti-inflammatory cytokines was significantly increased in PD-Ls-high tumors (Figure 6D). Cytokines were previously shown to be the positive regulators of HCC progression, including CXCL5, IFNG, CCL5, PLAU, PTN, CSF1, and HGF, and they were significantly up-regulated in tumors with higher PD-L1 or PD-L2 expression (43–49).There are also other cytokines inhibiting the progression of HCC, including IL15, IL18, and CD40LG. Our findings indicated there are conflicts between pro- and anti-inflammatory cytokines in promoting HCC progression. Meanwhile, the expression of regulatory T cell (Treg) markers was also up-regulated in tumors with higher expression of PD-Ls (Figures S7A,B). Evaluation of other immune checkpoints pathways showed that multiple inhibitory checkpoint molecules (except for ADORA2A) including PD-1 were at statistically higher levels in tumors with higher PD-L1 or PD-L2 expression (Figures S8A,B). These data suggested that the cooperation between PD-1 axis and other immune checkpoint pathways are common in mediating peripheral tolerance and immune escape in HCC.

### Tissue-Infiltrating Immune/Stromal Cell Populations in HCC Tumors With High vs. Low PD-Ls Expression

**Figure 7:**
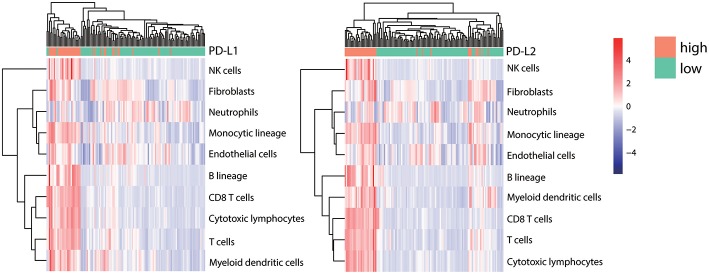
Correlation between PD-Ls and immune/stromal cell infiltration. The proportions of infiltrating immune and stromal cells between HCC subsets stratified by PD-L1 (left panel) and PD-L2 (right panel) expression. Unsupervised clustering shows the consistency between TILs infiltrating patterns and candidate grouping based on PD-Ls expression.

It has been hypothesized that within the tumor microenvironment, the recognition of cancer-associated antigens by CD8^+^ T cells can lead to the local production of IFN-γ, which ultimately induces the adaptive expression of PD-L1 on neighboring tumor infiltrating immune cells and tumor cells (5, 50–52). To analyze the correlation between immune cell infiltration and PD-Ls expression in HCC, the abundance of tissue-infiltrating immune and non-immune stromal cell populations in patients with different expression of PD-Ls (PD-L1 low vs. high; PD-L2 low vs. high) were evaluated by using the R package MCPcounter [Supplementary Materials; (53)]. A distinct distribution of immune and stromal cell types was found between different HCC subtypes, in which tumors with higher PD-L1 or PD-L2 expression had more abundant infiltrating immune cells, including natural killer (NK)-cells, monocytes, B cells, different types of T cells, dendritic cells, and certain types of stromal cells (fibroblasts and endothelial cells) (Figure 7). In order to determine the association of PD-Ls with immune cell function, we analyzed the enrichment data of six immune function gene sets as reported previously (36). The results showed that PD-L1 (Figure S9A) demonstrated a strong correlation with co-inhibition by antigen presenting cells (APC, Cor = 0.494), while PD-L2 (Figure S9B) demonstrated strong correlations with: (i) co-inhibition by APCs (Cor = 0.620); (ii) co-inhibition by T cells (Cor = 0.545), and (iii) co-stimulation by T cells (Cor = 0.512).

## Discussion

The roles of PD-Ls in predicting survival outcomes of HCC patients remains controversial. Gao et al. reported that higher expressions of PD-L1 and PD-L2 in tumor cells were associated with poorer survival outcomes of HCC patients. However, the study of Sideras et al. showed that PD-L1 expressed by tumor cells correlates with superior survival outcomes (54). In this study, we found that the higher expression of PD-L1 in HCC stroma was associated with poorer survival outcomes. It is worth noticing that previous studies have mainly focused on the association between the expression of PD-Ls in HCC tumor cells and survival outcomes. While positive PD-L1 staining could also be observed in HCC tumor cells, it's hard to distinguish positive stainings on membranes from those in cytoplasms, which was consistent with the report from previous study (18). Thus, no positive PD-L1 staining was recorded due to the methods used in our work (Materials and Methods). Therefore, only PD-L1-positive status in HCC tumor stroma has been recorded. On the other hand, the expression of PD-L2 could be detected in both HCC tumor cells and immune stroma. In addition to the fact that the frequency of PD-L2 expression is higher than PD-L1 in tumor cells (55), our findings are more clinically relevant because anti-PD-L1 treatment alone has a lower response rate than anti-PD-1 (12 and 27%, respectively) (16, 56). While no statistically significant correlation was found between PD-L2 expression in immune stroma and OS/DFS outcomes (Figure 1D, lower panel), two subgroups of patients with different survival outcomes based on PD-L2 in stroma can be easily identified from the survival curves. Considering statistically significant correlation of PD-L1 with OS/DFS (Figure 1D, upper panel), this result might be partially owing to the discordance between PD-L1 and PD-L2 expressions in over 40% of the patients included. In order to confirm that the antibodies used in this study could provide correct staining pattern in IHC tissues, placental tissue was used as a positive control. The results demonstrated the selective PD-L1 and PD-L2 staining of the syncytiotrophoblast layer in placental tissue, validating the excellent status of antibodies in all of our experiments (Figure S10). Therefore, our results play a pivotal role in ending the controversy surrounding the different distributions of PD-L1 and PD-L2 in the HCC tumor microenvironment.

It is well-established that the development and progression of cancer is regulated by genetic and epigenetic alterations in the transformed cells. However, many steps in cancinogenesis, including proliferation, invasion and angiogenesis, have been proven to be manipulated by tumor stroma, which consists of cancer associated fibroblasts (CAFs), tumor endothelial cells (TECs) and tumor-associated macrophages (TAMs) (57). On the other hand, while most cancer research has focused on the mutations in oncogenes or tumor suppressor genes that promote cancer formation, it has been widely perceived that the non-malignant cells in the tumor microenvironment progress along with the tumor cells and can provide support for their malignant phenotype (58). Among the normal cells in the tumor microenvironment, macrophages have been demonstrated to be the most abundant (59). Rather than exhibiting antitumor effects after being activated *in vitro*, macrophages acquire a protumoral phenotype *in vivo* (60, 61). These facts have highlighted the immune function of macrophages in cancer immune stroma and the importance of understanding their roles and potential mechanisms in tumor immunopathogenesis. For the first time, our study observed the co-existence of PD-L1 and PD-L2 in macrophages within HCC immune stroma. Our results also showed that both PD-L1 and PD-L2 in immune stroma are independent prognostic factors for OS along with the well-established factors including TNM stage and tumor size. These findings strongly suggest the pro-oncogenic role of macrophages in HCC immune stroma.

**Figure 8:**
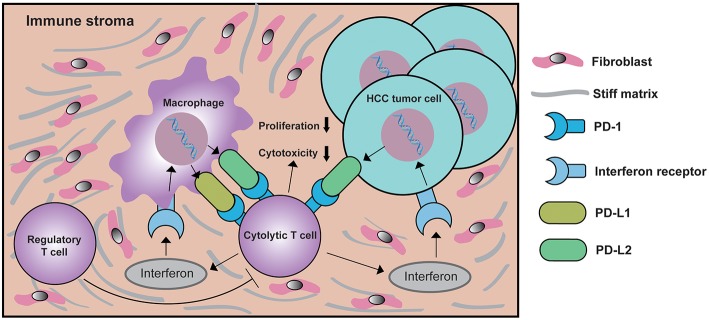
Schematic depiction of the mechanisms underlying PD-Ls-mediated cytolytic T cell exhaustion in the HCC microenvironment. In response to interferon γ signaling activated by cytolytic T cell, macrophages in immune stroma up-regulate PD-L1 and/or PD-L2 expression. At the same time, the expression of PD-L2 on HCC tumor cell membranes is also increased. The ligation of PD-Ls in macrophage and HCC tumor cells with PD-1 will down-modulate the function of cytolytic T cells, eventually creating a negative feedback loop that inhibits the proliferation and cytotoxicity of cytolytic T cells, thus reducing antitumor immunity. During this process, the function of regulatory T cell (Treg) is enhanced, which further suppresses the function of cytolytic T cells.

Currently, the association between the expression of PD-Ls and tumor infiltrating lymphocytes (TILs) has been reported in various cancers (52, 62, 63). Expression of PD-L1 on tumor cells or macrophages can reduced Granzyme B and IFN-γ expressed by CD8^+^ T cells, leading to the dysfunction of cytolytic activity in CD8^+^ T cells (64). Considering the overlapping functions of PD-L1 with PD-L2, this fact highly suggests that both PD-L1 and PD-L2 regulate cytolytic functions of CD8^+^ T cells. Although the infiltration of CD8^+^ TILs in HCC tissue has been reported, no study has ever focused on the correlation between CD8^+^ TILs and the expression of PD-Ls in HCC immune stroma ever since the co-existence of CD8 and PD-L1 in LEL-HCC was validated (20). Therefore, research on the correlation between CD8^+^ TILs and PD-Ls is of great significance. Consistent with previous studies (32), small clusters of CD8^+^ TILs were observed in HCC immune stroma. At the same time, the expression of PD-Ls positively correlated with CD8^+^ TIL infiltration. Further supported by the facts that both PD-L1 and PD-L2 were predominantly expressed by immune stromal cells from our study, we believe there is a strong correlation between the expression of PD-Ls and the infiltration of CD8^+^ TILs in HCC immune stroma. After stratifying patients into different subgroups according to the expression of PD-Ls and CD8, we were able to identify patients with PD-Ls-negative status and CD8^+^ TIL infiltration, suggesting other suppressor(s) could be involved in promoting immune tolerance (type IV tumors) according to the classification method raised by Teng et al. (65). The aforementioned patients have the best survival outcomes. Patients have the worst prognosis when showing expression of PD-Ls but no TIL infiltration, indicating the presence of tumors with oncogenic pathways inducted by PD-Ls (type III tumors). Considering that macrophages are the predominant immune cell subtype expressing both PD-L1 and PD-L2, we propose a new mechanism of how macrophages regulate PD-Ls, during the progression of HCC, macrophages in immune stroma up-regulate PD-Ls in response to IFNγ released by TILs as an adaptive immune-resistance mechanism to suppress CD8^+^ effector T cell function, thus leading to CD8^+^ T cell exhaustion/inhibition and poorer survival outcomes (Figure 8).

PD-Ls' expression effect and the effect being impacted by anti-tumor immunity in the HCC microenvironment have not been systematically illustrated. Previous study on multiple tumor models demonstrated that, despite the accumulation of CD8^+^ T cells and NK cells during tumor progression, the activation of these immune cells will be substantially inhibited by the binding of PD-Ls to them, which attenuates the anti-tumor activity of these cells (64). For the first time, we used large-scale transcriptional and genetic data to explore the genomic landscape of HCC in the context of different expression levels of PD-Ls. By stratifying patients based on expression of PD-Ls, we found higher levels of PD-Ls are correlated with higher expression of cytokines, immune checkpoint molecules, Treg markers, and higher cytolytic activity. These results indicate that tumors highly expression PD-Ls are likely to possess pre-existing antitumor immunity. Therefore, they can subsequently arouse immune tolerance and initiate a feedback loop during the progression of HCC. On the other hand, the similarities between PD-L1 and PD-L2 in affecting CNV suggest their synergistic effect in regulating gene expression, potentially shaping the microenvironment within HCC tissue. Moreover, by using the R package MCPcounter, we found that, in HCC tissue, higher expression of PD-Ls was associated with a higher proportion of infiltration of immune cells, as well as stromal cells including fibroblasts and endothelial cells. Further validation by using Tumor Immune Estimation Resource (TIMER; cistrome.shinyapps.io/timer) (66) showed that a positive correlation between the expression of PD-Ls and tumor immune cell infiltration. However, a negative correlation between expression of PD-Ls and tumor purity is observed, suggesting that both PD-L1 and PD-L2 are expressed by non-cancerous cells mainly consisting of immune cells in the HCC tumor microenvironment (Figure S11). Combining these findings with the results obtained by immunohistochemistry, we can draw a conclusion that HCC tissue with a higher expression of PD-L1 or PD-L2 are affected and characterized by the existence of a substantial immune stroma consisting of various infiltrating immune cells. Taken together, our findings promote further understanding of the molecular pathology of PD-Ls in the HCC microenvironment.

## Conclusion

In conclusion, this study has highlighted the essential role of PD-Ls expressed by macrophages in HCC immune stroma and their potency in regulating the tumor microenvironment. In addition, this study has provided potentially practical management options for future clinical trials on anti PD-1 axis therapy for HCC patients, it should be based on the expression of PD-L1, PD-L2, and CD8 along with the clinicopathological characteristics of HCC patients. Further studies will focus on the blockade of both PD-L1 and PD-L2 in macrophages for the immunotherapy of HCC.

## Data Availability

Publicly available datasets were analyzed in this study. This data can be found here: https://portal.gdc.cancer.gov/

## Ethics Statement

This study was carried out in accordance with the recommendations of guideline in human sample research from the ethics committee of West China Hospital. Informed consent for the use of tissue for the purpose of biomedical research was provided by every individual patient, with the approval from the ethics committee of West China Hospital.

## Author Contributions

YZ, KY, and JG designed this study. HL and WC performed the experiments. HL performed statistical analysis and write the manuscript. YD, JR, and KX helped revising the manuscript. All authors read and approved the final manuscript.

### Conflict of Interest Statement

The authors declare that the research was conducted in the absence of any commercial or financial relationships that could be construed as a potential conflict of interest.

## Abbreviations

PD-L1: programmed cell death-ligand 1
PD-L2: programmed cell death-ligand 2
PD-1: programmed death receptor 1
HCC: hepatocellular carcinoma
TME: tumor microenvironment
IHC: immunohistochemistry
TCGA: The Cancer Genome Atlas
OS: overall survival
DFS: disease-free survival
CNV: copy number variation
HBV: hepatitis B virus
HCV: hepatitis C virus
FFPE: formalin-fixed paraffin-embedded
AFP: α-fetoprotein
IF: immunofluorescence
TPM: transcripts per million
CPM: count per million
SMGs: significantly mutated genes
BMR: background mutation rate
FDR: false discovery rate
WES: whole exome sequencing
GEO: Gene Expression Omnibus
HR: hazard ratio
LEL: lymphoepithelioma-like
CAFs: cancer associated fibroblasts
TECs: tumor endothelial cells
TAMs: tumor-associated macrophages
TILs: tumor infiltrating lymphocytes
IFNγ: interferon γ.
