# Supplementary material for: Expression of Programmed Cell Death-Ligands in Hepatocellular Carcinoma: Correlation With Immune Microenvironment and Survival Outcomes
Source: Front Oncol. 2019 Sep 11;9:883. doi: 10.3389/fonc.2019.00883 (PMC6749030; doi:10.3389/fonc.2019.00883)
Supplement: Supplementary file 1 [file Presentation_1.pdf]

Supplementary materials

Materials and Methods

RNAseq gene expression analysis

Raw counts of gene expression from RNAseq were downloaded from the TCGA data portal (https,//tcga-data.nci.nih.gov/) for the differential gene expression analysis. Total raw read counts per gene were divided by the gene's maximum transcript length to represent a coverage depth estimate, which were then scaled to a total depth of 10^6 per sample and can be interpreted as transcripts per million (TPM) (1). Statistical ranking for PD-L1 expression by the top decile and bottom quartile were defined as PD-L1-high and PD-L1-low, respectively. The same method was used to define the expression level of PD-L2. Differential gene expression analysis between PD-L1-high and PD-L1-low (or PD-L2-high and PD-L2-low) across TCGA datasets was calculated using the R package edgeR, which determines the differential gene expression by accounting for variability through an over-dispersed Poisson model and moderating the degree of over-dispersion by Empirical Bayes methods (2). Genes with counts per million (CPM) larger than 1 across at least 13 samples (10% of all samples) were included for differential gene expression analysis. Genes with the adjusted *p* value less than 0.05 and the absolute FC larger than 1.5 were considered to be statistically significant.

Mutation and copy number variation analysis

Significantly mutated genes (SMGs) were defined by running the Mutational Significance in Cancer (MuSiC Genome Suite) in different subtypes of HCC (PD-L1-high vs. PD-L1-low and PD-L2-high vs. PD-L2-low, respectively). MuSiC identifies genes with significantly higher mutation rates than the background mutation rate (BMR) to find SMGs across the entire sample population. The threshold for significance was a false discovery rate (FDR) of 0.1. Mutational spectra across the entire study population from the TCGA dataset were determined as previously described (1, 3). Copy number variation (CNV) data was downloaded from GDAC Firehose and separated into different datasets according to the expressions of PD-L1 and PD-L2. Investigation into significant amplification or deletion events in the regions of the genome was conducted through the use of GISTIC 2.0, a revised computational program to identify somatic copy number alteration by investigating the frequency and amplitude of observed events (4). Meanwhile, genes within the significant genomic regions were further analyzed to determine the overlay with those differentially expressed and identified from RNAseq.

Neoepitopes and cytolytic T-cell activity

The number of neoepitopes in each HCC subtype was referenced from the previous study (1). Briefly, normal tissue whole exome sequencing (WES) was used as input to determine Human Leukocyte Antigens (HLA) type for each sample by using POLYSOLVER (POLYmorphic loci reSOLVER). POLYSOLVER then identified HLA mutations by comparing to matched tumors. By defining all novel amino acid 9mers and 10mers resulting from mutation in expressed genes (median >10 TPM in the tumor type) and determining whether the predicted binding affinity to

the patient's germline HLA alleles was smaller than 500 nM using NetMHCpan (5), neoepitopes for each patient can be predicted. The measure of immune cytolytic activity is based on transcript levels of two key cytolytic effectors, granzyme A (GZMA) and perforin (PRF1), which are dramatically upregulated upon CD8+ T cell activation (6) and during productive clinical responses to anti-CTLA-4 and anti-PD-L1 immunotherapies (7, 8).

Tissue-infiltrating immune and stromal cell estimation

Estimation on the population abundance of tissue-infiltrating immune and stromal cell populations in HCC tissue was performed by the R package MCPcounter using the RNAseq data from TCGA (9). Scores from RNAseq data are proportional to the cells of a certain population within the whole sample and cells that are expressed in arbitrary units specific for each population, including $CD3^{+}$ T cells, $CD8^{+}$ T cells, cytotoxic lymphocytes, NK cells, B lymphocytes, cells originating from monocytes (monocytic lineage), myeloid dendritic cells, neutrophils, endothelial cells and fibroblasts.

Gene Expression Omnibus (GEO) data

Microarray gene expression data of HCC samples were downloaded from the GEO database (accession numbers GSE54236, GSE64041) (10, 11). The R package "GEOquery" was used to extract the expression values of genes.

Figures

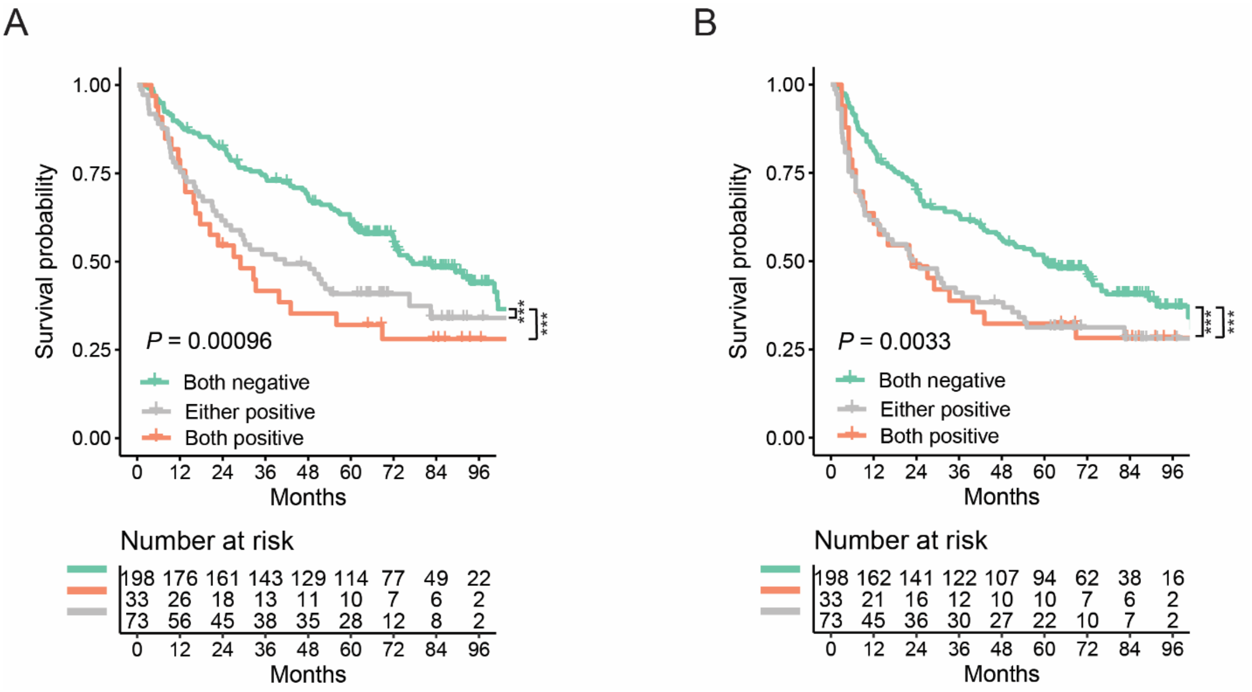

Figure S1. The association of combined PD-Ls expression in immune stroma with OS (A) and DFS (B) outcomes (Log-rank test). ***, P <0.001.

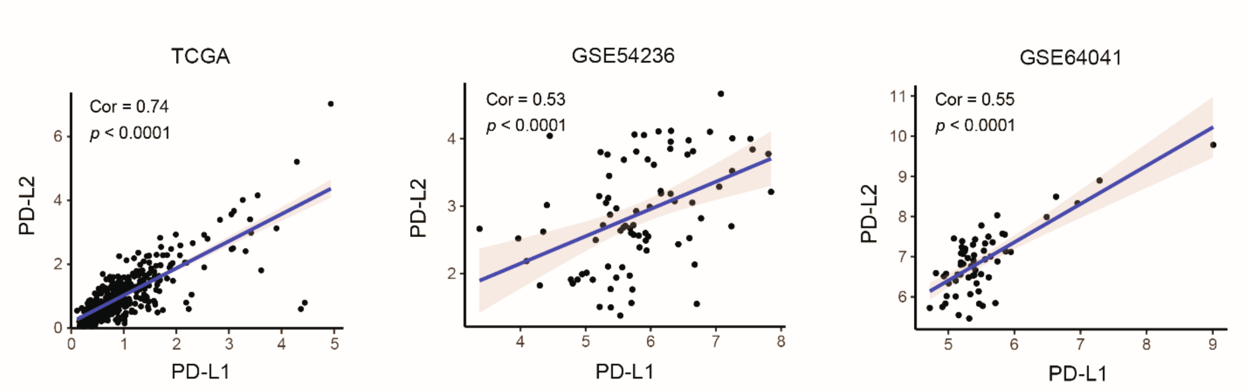

Figure S2. Correlation between the mRNA expression levels of PD-L1 and PD-L2 in TCGA and GEO datasets.

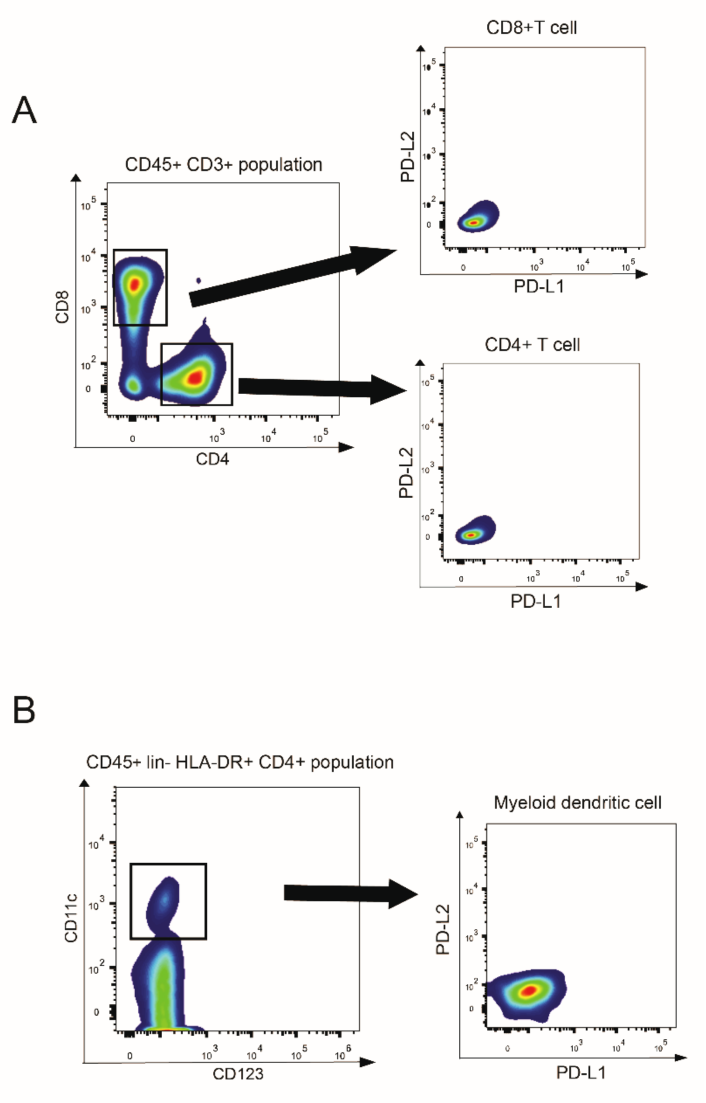

Figure S3. Absence of PD-Ls in T cells (A) and myeloid dendritic cells (B).

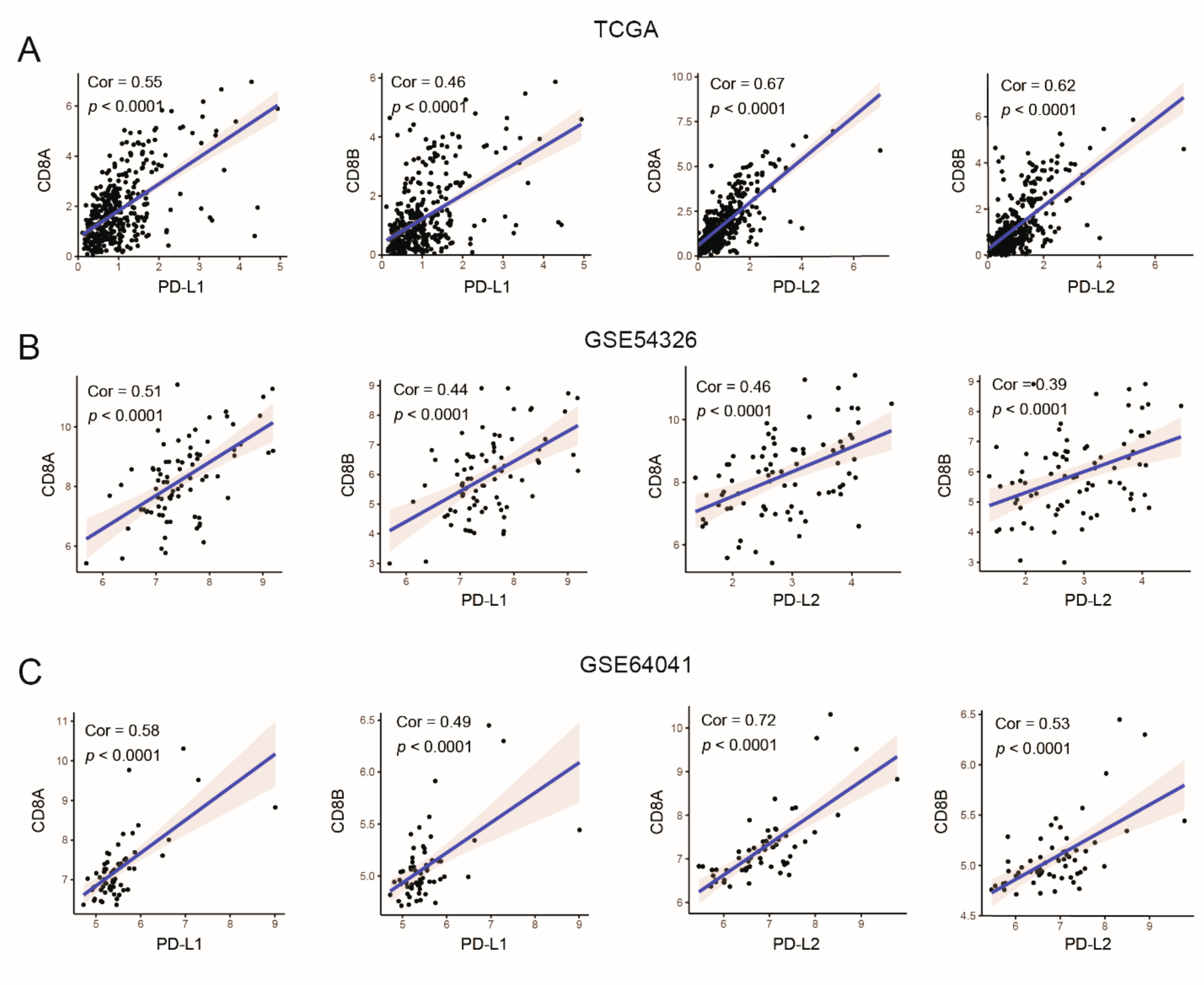

Figure S4. The correlation between PD-Ls and CD8 expression in TCGA (A) and GEO (B-C) datasets.

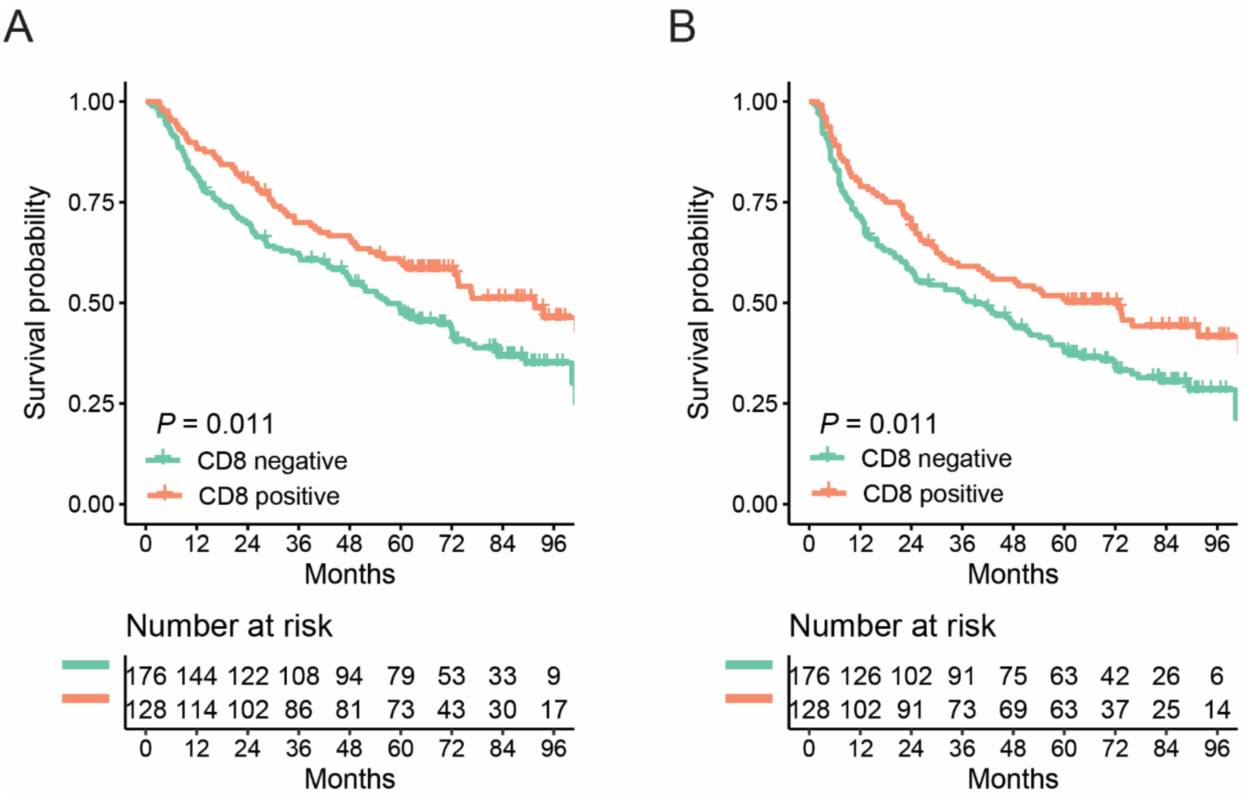

Figure S5. Correlation of CD8 expression with OS(A) and DFS(B) outcomes.

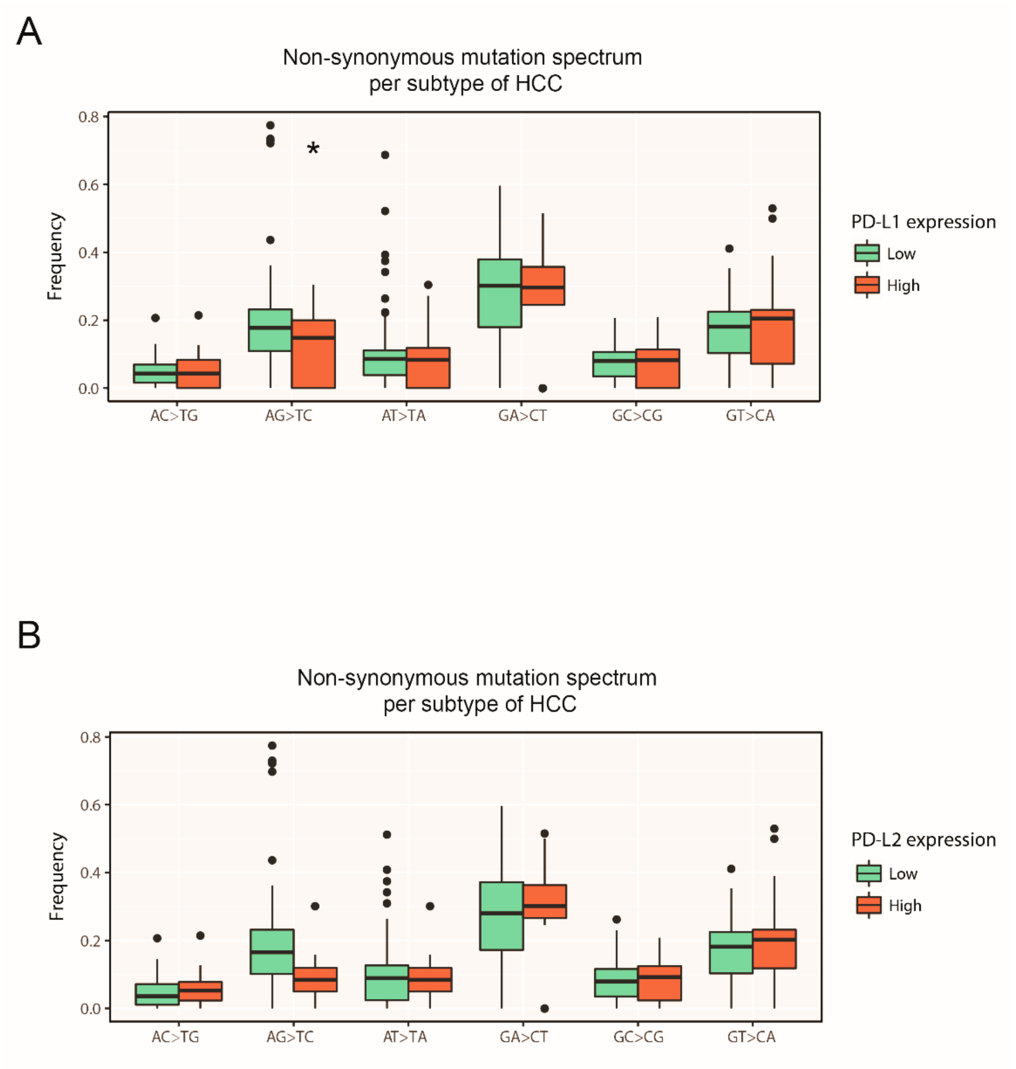

Figure S6. The frequency of specific substitutions between PD-L1-high and -low tumors (A) or between PD-L2-high and -low tumors (B).

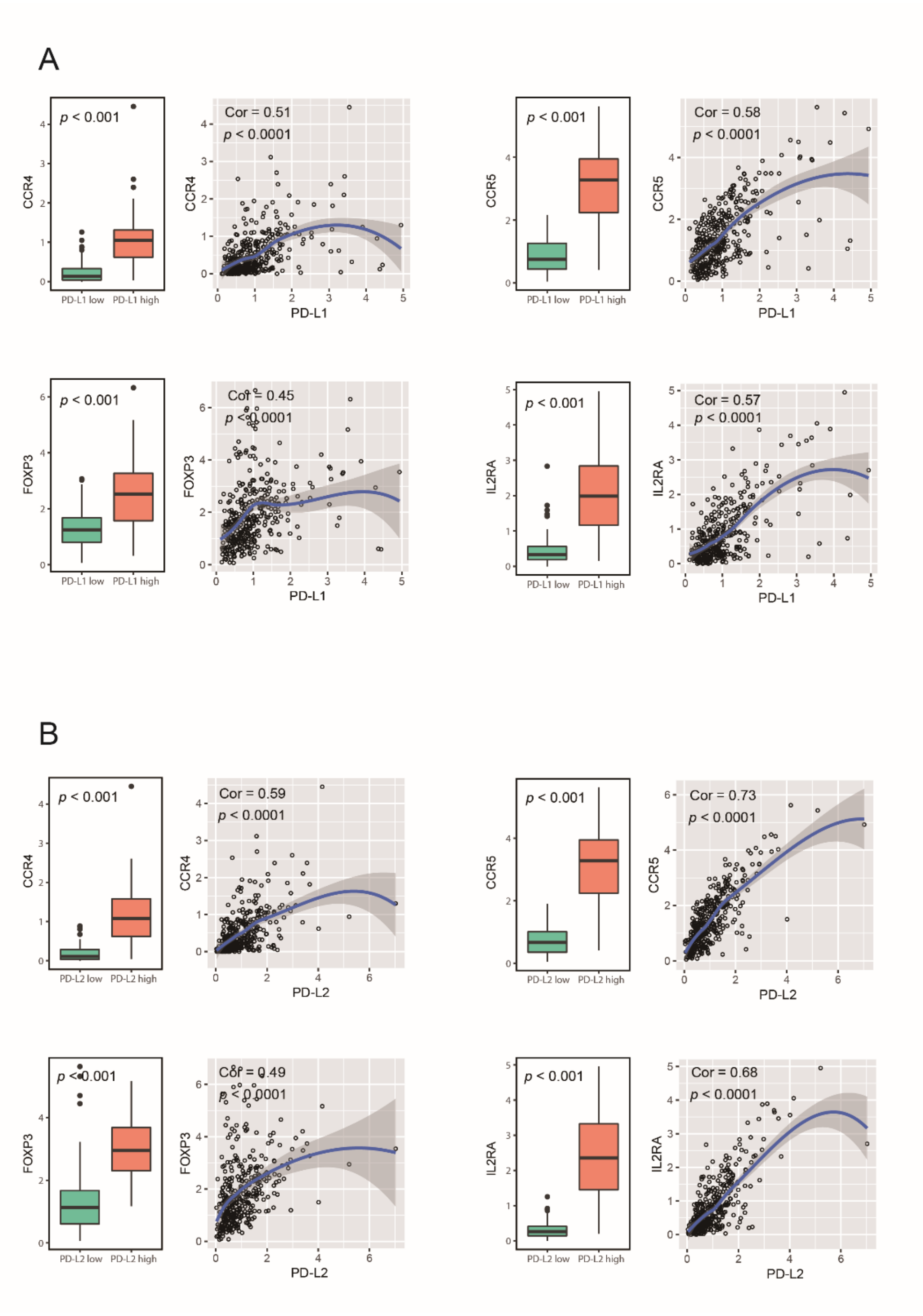

Figure S7. the expression of regulatory T cell (Treg) markers was also up-regulated in tumors with higher expression of PD-L1 (A) or with higher expression of PD-L2 (B).

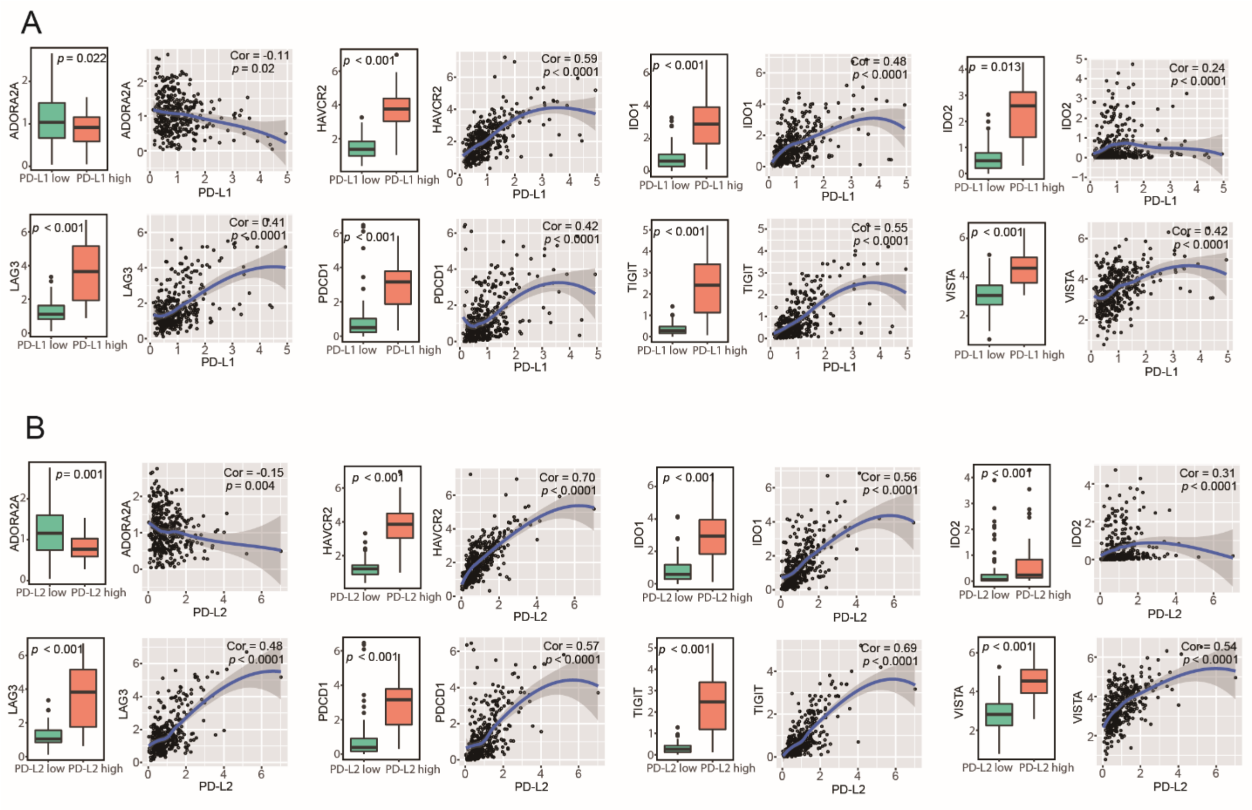

Figure S8. Evaluation of other immune checkpoints molecules between PD-L1-high and -low tumors (A) or between PD-L2-high and -low tumors (B).

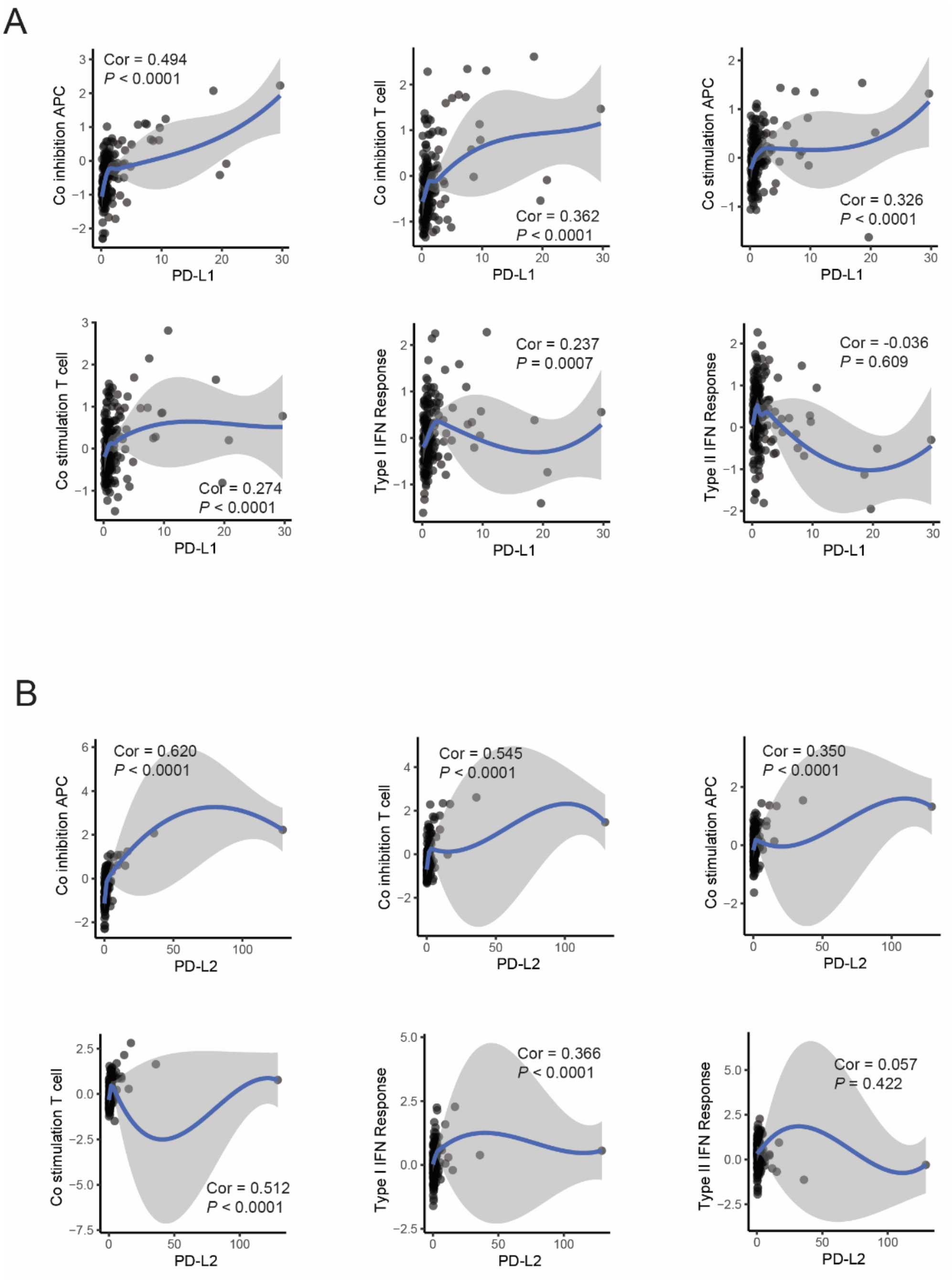

Figure S9. Local regression curves showing the correlation of PD-L1 (A) and PD-L2 (B) expressions with the function of different immune cells (Spearman rank correlation).

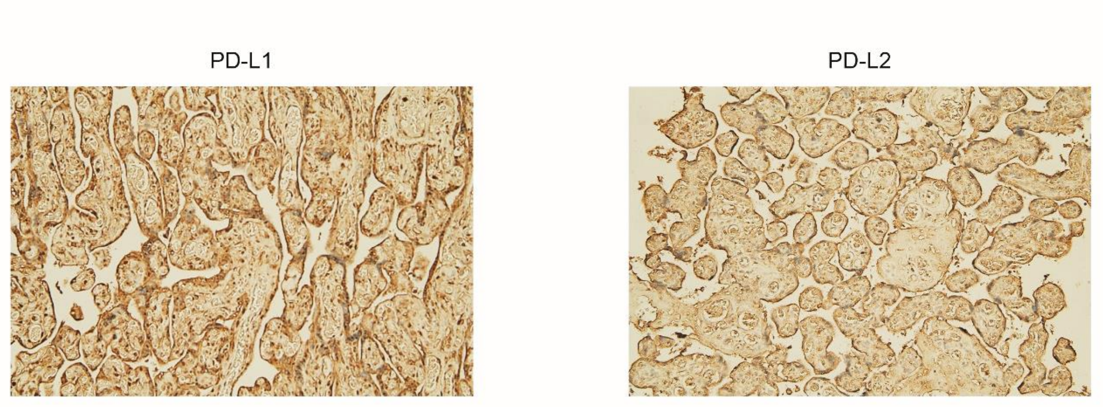

Figure S10. Positive controls for PD-L1 and PD-L2 staining.

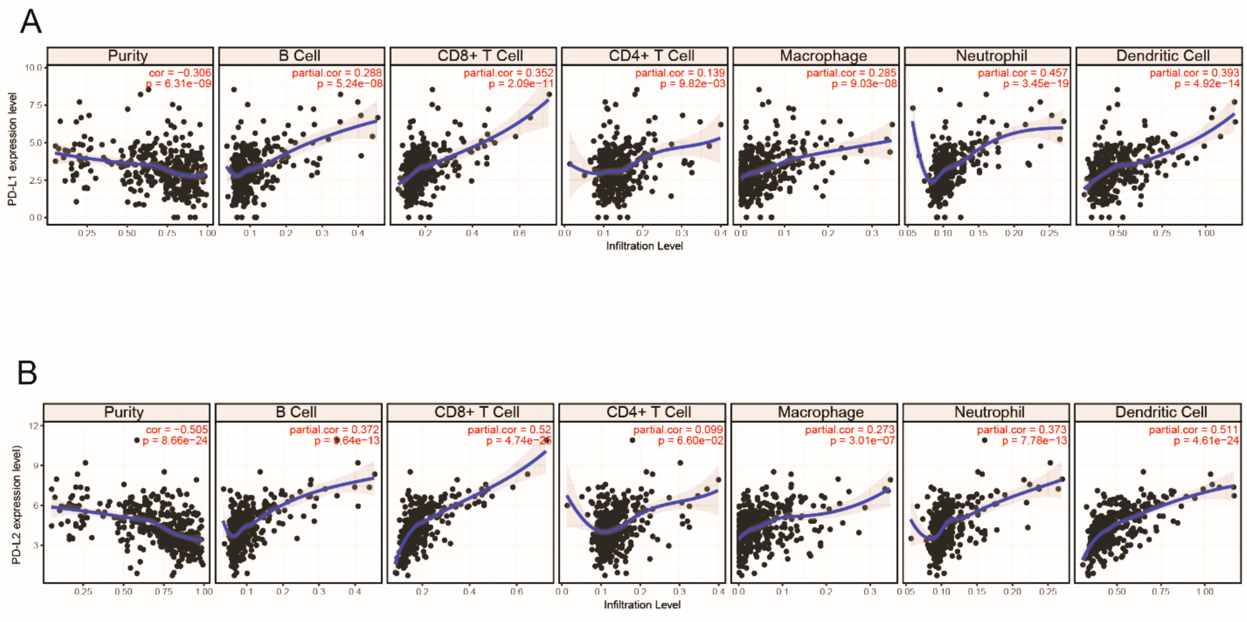

Figure S11. Association of PD-Ls (A, PD-L1; B, PD-L2) with tumor immune cell infiltration and purity in TIMER analysis.

References

1. Rooney MS, Shukla SA, Wu CJ, Getz G, Hacohen N. Molecular and genetic properties of tumors associated with local immune cytolytic activity. Cell. 2015;160(1-2):48-61.
2. Robinson MD, McCarthy DJ, Smyth GK. edgeR: a Bioconductor package for differential expression analysis of digital gene expression data. Bioinformatics (Oxford, England). 2010;26(1):139-40.
3. Comprehensive and Integrative Genomic Characterization of Hepatocellular Carcinoma. Cell. 2017;169(7):1327-41.e23.
4. Mermel CH, Schumacher SE, Hill B, Meyerson ML, Beroukhim R, Getz G. GISTIC2.0 facilitates sensitive and confident localization of the targets of focal somatic copy-number alteration in human cancers. Genome biology. 2011;12(4):R41.
5. Nielsen M, Lundegaard C, Blicher T, Lamberth K, Harndahl M, Justesen S, et al. NetMHCpan, a method for quantitative predictions of peptide binding to any HLA-A and -B locus protein of known sequence. PloS one. 2007;2(8):e796.
6. Johnson BJ, Costelloe EO, Fitzpatrick DR, Haanen JB, Schumacher TN, Brown LE, et al. Single-cell perforin and granzyme expression reveals the anatomical localization of effector CD8+ T cells in influenza virus-infected mice. Proceedings of the National Academy of Sciences of the United States of America. 2003;100(5):2657-62.
7. Herbst RS, Soria JC, Kowanetz M, Fine GD, Hamid O, Gordon MS, et al. Predictive correlates of response to the anti-PD-L1 antibody MPDL3280A in cancer patients. Nature. 2014;515(7528):563-7.
8. Ji RR, Chasalow SD, Wang L, Hamid O, Schmidt H, Cogswell J, et al. An immune-active tumor microenvironment favors clinical response to ipilimumab. Cancer immunology, immunotherapy : CII. 2012;61(7):1019-31.
9. Becht E, Giraldo NA, Lacroix L, Buttard B, Elarouci N, Petitprez F, et al. Estimating the population abundance of tissue-infiltrating immune and stromal cell populations using gene expression. Genome biology. 2016;17(1):218.
10. Makowska Z, Boldanova T, Adametz D, Quagliata L, Vogt JE, Dill MT, et al. Gene expression analysis of biopsy samples reveals critical limitations of transcriptome-based molecular classifications of hepatocellular carcinoma. The journal of pathology Clinical research. 2016;2(2):80-92.
11. Villa E, Critelli R, Lei B, Marzocchi G, Camma C, Giannelli G, et al. Neoangiogenesis-related genes are hallmarks of fast-growing hepatocellular carcinomas and worst survival. Results from a prospective study. Gut. 2016;65(5):861-9.
